# Modulation of Neuro-Inflammatory Signals in Microglia by Plasma Prekallikrein and Neuronal Cell Debris

**DOI:** 10.3389/fphar.2021.743059

**Published:** 2021-11-15

**Authors:** Aneese A. Jaffa, Miran A. Jaffa, Mayssam Moussa, Ibrahim A. Ahmed, Mia Karam, Kawthar Sharaf Aldeen, Rola Al Sayegh, Ghewa A. El-Achkar, Leila Nasrallah, Yara Yehya, Aida Habib, Fuad N. Ziyadeh, Ali H. Eid, Firas H. Kobeissy, Ayad A. Jaffa

**Affiliations:** ^1^ Department of Biology, Faculty of Arts and Sciences, American University of Beirut, Beirut, Lebanon; ^2^ Epidemiology and Population Health Department, Faculty of Health Sciences, American University of Beirut, Beirut, Lebanon; ^3^ Department of Biochemistry and Molecular Genetics, Faculty of Medicine, American University of Beirut, Beirut, Lebanon; ^4^ INSERM-UMR1149, Centre de Recherche sur l’Inflammation, and Sorbonne Paris Cité, Laboratoire d’Excellence Inflamex, Faculté de Médecine, Universite de Paris, Paris, France; ^5^ Department of Internal Medicine, Faculty of Medicine, American University of Beirut, Beirut, Lebanon; ^6^ Department of Basic Medical Sciences, College of Medicine, QU Health, Qatar University, Doha, Qatar; ^7^ Biomedical and Pharmaceutical Research Unit, QU Health, Qatar University, Doha, Qatar

**Keywords:** neuroinflammation, cytokines, plasma kallikrein-kinin system, protease-activated receptor 2, bradykinin 2 receptor, interactome

## Abstract

Microglia, the resident phagocytes of the central nervous system and one of the key modulators of the innate immune system, have been shown to play a major role in brain insults. Upon activation in response to neuroinflammation, microglia promote the release of inflammatory mediators as well as promote phagocytosis. Plasma prekallikrein (PKall) has been recently implicated as a mediator of neuroinflammation; nevertheless, its role in mediating microglial activation has not been investigated yet. In the current study, we evaluate the mechanisms through which PKall contributes to microglial activation and release of inflammatory cytokines assessing PKall-related receptors and their dynamics. Murine N9-microglial cells were exposed to PKall (2.5 ng/ml), lipopolysaccharide (100 ng/ml), bradykinin (BK, 0.1 μM), and neuronal cell debris (16.5 μg protein/ml). Gene expression of bradykinin 2 receptor (B_2_KR), protease-activated receptor 2 (PAR-2), along with cytokines and fibrotic mediators were studied. Bioinformatic analysis was conducted to correlate altered protein changes with microglial activation. To assess receptor dynamics, HOE-140 (1 μM) and GB-83 (2 μM) were used to antagonize the B_2_KR and PAR-2 receptors, respectively. Also, the role of autophagy in modulating microglial response was evaluated. Data from our work indicate that PKall, LPS, BK, and neuronal cell debris resulted in the activation of microglia and enhanced expression/secretion of inflammatory mediators. Elevated increase in inflammatory mediators was attenuated in the presence of HOE-140 and GB-83, implicating the engagement of these receptors in the activation process coupled with an increase in the expression of B_2_KR and PAR-2. Finally, the inhibition of autophagy significantly enhanced the release of the cytokine IL-6 which were validated via bioinformatics analysis demonstrating the role of PKall in systematic and brain inflammatory processes. Taken together, we demonstrated that PKall can modulate microglial activation via the engagement of PAR-2 and B_2_KR where PKall acts as a neuromodulator of inflammatory processes.

## Introduction

Neuro-inflammation is the inexorable, yet substantial, inflammation of the tissues of the central nervous system (CNS) mediated by the production of excessive cytokines, chemokines, and secondary messengers (DiSabato et al., 2016). This pathological process is initiated in response to a plethora of diverse cues, the most principal being traumatic brain injury (TBI), infection, or ischemic injury, all of which are types of damages to the CNS (DiSabato et al., 2016). Pro-inflammatory cytokine inducers, such as lipopolysaccharide (LPS), as well as contact with neuronal cell debris, have been shown to typically activate microglia (Lenz and Nelson, 2018). Activated microglia are involved in the production of inflammatory cytokines and mediators, which may promote tissue inflammation and damage (Bachiller et al., 2018).

Microglia, repeatedly described as the resident mononuclear phagocytes of the CNS and the primary reactors towards brain injury, responding by induction of immunological activities or repair of tissue injury (Chen et al., 2012). In the resting state microglia function as supportive glial cells, however, upon encounter with injured brain tissue or cellular debris, microglia become activated, commonly indicated by morphological and inflammatory gene expression changes, allowing for more innate immune cell responses not previously present in the inactive state (Vilhardt, 2005; Chen et al., 2012; Wang et al., 2015). Recent studies demonstrate that phagocytosis is a quintessential process by which the microglial cells promote the clearance of apoptotic and injured cells in the CNS (Janda et al., 2018; Yanuck, 2019) and contribute to the overall brain function during disease and homeostatic states (Yanuck, 2019).

Plasma prekallikrein (PKall) is a multifunctional serine protease and its substrate high molecular weight kininogen (*KNG*) have been linked to the activation of the intrinsic coagulation pathway resulting in thrombus formation (Bjorkqvist et al., 2013; Weidmann et al., 2017). Traditionally, PKall has been shown to mediate its effects via the cleavage of *KNG* to release the pro-inflammatory peptide bradykinin (BK), which in turn acts on bradykinin 2 receptors (B_2_KR) to mediate its effects (Terzuoli et al., 2014; Dagher et al., 2019; Al Hariri et al., 2020; Saoud et al., 2020). However, it has been recently discovered that PKall can transduce its signals utilizing direct interaction and activation of protease-activated receptors 1 (PAR 1) and 2 (PAR 2), both of which are members of the G-protein coupled receptors (Abdallah et al., 2010; Heuberger and Schuepbach, 2019).

Although PKall was shown to modulate brain endothelial cells in neuroinflammation via engagement of PAR2 (Gobel et al., 2019), its role in modulating the activation of microglia has not being explored. Therefore, in the current study, we evaluated whether microglial cells express components of the plasma prekallikrein-kinin system (PKKS) and assessed the factors that modulate their expression. In addition, we delineated the mechanisms through which PKall promotes inflammatory signals and activation of microglial cells utilizing both biochemical approaches substantiated with bioinformatics approaches.

## Methods and Materials

### Induction of Apoptosis in PC12 Cells

To generate apoptotic neuronal cell debris (NCD), PC12 (PC-12 ATCC CRL-1721) cells were treated with 100 nM Staurosporine (569397-100UG, Calbiochem) for 24 h to induce their apoptosis (Simone et al., 2003; Nokkari et al., 2015). Post Staurosporine treatment, PC12 media containing the detached cells was collected and then centrifuged at 14,000 g and the resulting pellet was washed three times with PBS, and then dissolved in 100 μl PBS. As to the attached apoptotic PC12 cells, they were washed three times with PBS to remove any residual Staurosporine, extracted from the well plates and then centrifuged at 14,000 g resulting in a pellet constituting the neuronal cell debris which was dissolved in 200 μl PBS and mixed with the pellet from the media (100 μl). Protein assay (BIO-Rad) was used to determine protein concentration and the resulting combined apoptotic NCD were used in our experiments to activate the microglial cells.

### Cell Culture and Treatment

Murine N9 microglial cells (a generous gift from EnCor biotechnology, Gainesville, FL, United States (https; //encorbio.com/) were utilized in our study. The N9 microglial cells are derived from mouse brain and shares many phenotypical characteristics with primary mouse microglia (Hickman et al., 2008). They are immortalized with the v-myc or v-mil oncogenes of the avian retrovirus, and are highly compatible for answering specific inflammatory research questions as they are highly characterized with proliferation and adherence compared to the primary microglial cells that result in low cell number and require time consuming techniques (Stansley et al., 2012).

The microglial cells were cultured in 12-well plates in Dulbecco’s Modified Eagles Medium/Nutrient Mixture (DMEM) F-12 Ham medium (Sigma-Aldrich, United Kingdom) supplemented with 10% fetal bovine serum (FBS), 100 IU/ml penicillin, and 100 μg/ml streptomycin, incubated at 37°C in the presence of 5% CO_2_. To achieve quiescence (80% confluence), microglia cells were grown in DMEM media containing 2% FBS for 24 h. Following this, microglia cells were stimulated with LPS (100 ng/ml, diluted in water), a component of the Gram-negative bacterial cell known to induce inflammatory cytokines as a positive control, PKall (2.5 ng/ml, diluted in water), BK (0.1 μM, diluted in 0.1 M acetic acid), and NCD (16.5 μg protein/ml) for 24 h to assess their effects on the expression of inflammatory cytokines and activation of microglia cells. The concentration of LPS (100 ng/ml) was determined from the literature from studies assessing the effects of LPS on microglial cells (He et al., 2021). The Concentration of BK (0.1 µM) was selected based on our prior studies assessing the effects of various concentrations of BK on signaling pathways in different cells (Dagher et al., 2019; Al Hariri et al., 2020; Saoud et al., 2020). The concentration of PKall was determined from concentration-response curves assessing different concentrations of PKall (0, 1.0, 2.5, 5.0, 7.5, and 10 ng/ml) on ERK1/2 phosphorylation. The peak response was determined at 2.5 ng/ml. The concentration of NCD that was used in our studies was determined from pilot experiments in which microglial cells were exposed to various concentrations (0, 11, 16.5, 27.5, and 55 µg protein/ml) of NCD for 24 h. The mRNA levels of IL-6 and TNF-α measured in response to the different concentrations of NCD, indicated that the response plateaued at 16.5 µg protein/ml.

To identify what receptors are involved, microglia cells were pretreated for 30 min with a B_2_KR antagonist HOE140 (1 μM) and/or a PAR2 antagonist GB83 (2 μM, Axon Medchem BV, Cedarlane), followed by treatment with PKall, BK, and neuronal cell debris. Following a 24-h treatment period, cell media was collected for measurement of cytokines by ELISA, and the cells were used to extract proteins or mRNA. Changes in the microglial cell morphology in response to LPS, PKall, BK, and neuronal cell debris were assessed by confocal microscopy.

### RNA Extraction and Real-Time Polymerase Chain Reaction

Total RNA from N9 microglia cells was extracted using RiboZol reagent (Amresco) according to the manufacturer’s protocol. Total RNA concentration was determined by Nanodrop 1,000 (Thermo Scientific) and the 260/280 ratio was evaluated. The resulting RNA was reverse transcribed into cDNA followed by amplification of the cDNA using the iQ SYBR green supermix kit (Bio-Rad) as previously described (Al Hariri et al., 2020). The following forward and reverse murine gene primers were used (Macrogen Inc. Seoul, South Korea): GAPDH (Forward: AAA GTC GCG TGA TGG CCG, Reverse: CGA CGG ACA CAT TGG GGG TAG GA); IL-6 (Forward: GGA GTG GCT AAG GAC CAA GAC, Reverse: GCA TAA CGC ACT AGG TTT GCC); B_2_KR (Forward: TCA​ACT​GCC​CAG​ACA​CTG​AG, Reverse: CAG​AAC​ACG​CTG​AGG​ACA​AA); PAR2 (Forward: TGC​TTT​GCT​CCT​AGC​AAC​CT, Reverse: CAG​AGG​GCG​ACA​AGG​TAG​AG); LGALS3 (Forward: ACT​GCC​CTG​GAC​ACC​AAT​AG, Reverse: TAG​AAG​GGG​CGT​ATG​ACC​AC); KNG (Forward: GCC​AGG​GAG​CAA​GAA​GAG​AG, Reverse: CCC​ATG​CTT​ATG​ACC​ACG​GT); COX2 (Forward: TTG​GAG​GCG​AAG​TGG​GTT​TT, Reverse: GGT​AGG​CTG​TGG​ATC​TTG​CA); IL-1β (F: GCT​GCT​TCC​AAA​CCT​TTG​AC, Reverse: TGT​CCT​CAT​CCT​GGA​AGG​TC); TNF-α (F: CGT​CAG​CCG​ATT​TGC​TAT​CT, Reverse: CGG​ACT​CCG​CAA​AGT​CTA​AG); PKall (Forward: CCC​ATG​GAT​ATT​TTC​CAG​CA, Reverse: AGA​TGG​TGC​GAC​ACA​CAA​AG). The mRNA levels of expressed genes were calculated using the 2^−ΔΔCt^ calculation formula and expressed relative to GAPDH mRNA as a reference gene.

### Enzyme-Linked Immunosorbent Assay

The levels of TNF-α and IL-6 were measured using ELISA (Thermo Fisher Scientific, Waltham, MA, United States) in the culture media of the microglial cells treated with LPS, PKall, BK, and neuronal cell debris according to the manufacturer’s instructions. The optical density of each sample was read at 450 nm.

### Cell Viability Assay

Microglial cell viability was determined by the MTT (Thiazolyl Blue Tetrazolium Bromide) reduction assay dye (Sigma-Aldrich, United States, M5655) in response to LPS, BK, PKall, and NCD treatment for 24 h. Following this, the media was discarded and 90 µl of serum-free media along with 10 µl of MTT solution was added into each well and incubated for 3 h at 37°C. At the end of the incubation period, 150 µl of MTT solvent was then added into each well and incubated for 15 min before the absorbance was read at an optical density of 590 nm (Thermo Scientific Multiskan EX, Model 355).

### Immunofluorescence Staining

Microglial cells cultured on coverslips in 2% FBS were stimulated with LPS (100 ng/ml), BK (0.1 μM), PKall (2.5 ng/ml), and NCD (16.5 μg protein/ml) for 24 h. Following this, the supernatant was discarded and the microglial cells were fixed with 4% paraformaldehyde for 30 min, and then washed with phosphate-buffered saline (PBS), followed by a 3-time wash with PBST (PBS +0.1% Triton X-100). A blocking solution of 10% FBS in PBST was then added and the plate was incubated for 1 h. Cells were then incubated with primary polyclonal anti-coronin antibodies (1/1,000 dilution) and anti-IBA-1 antibodies, a selective antibody for macrophage and microglial cells (1/1,000 dilution, Encor Biotechnologies, catalogue # AB-2722474) for 24 h at 4°C. After washing, the cells were incubated with the corresponding secondary antibodies Alexa-fluor 488 and/or 568 conjugated IgG for 2 h at 4°C. The nucleus was stained with 4′, 6-diamidino-2-phenylindole (DAPI). Immunofluorescence images were visualized using a laser scanning confocal microscope at 63x magnification (Leica Microsystems, Cambridge, United Kingdom). Staining intensity and cell diameter were calculated using Zen software (Carl Zeiss, Germany2.7). Detection of coronin and IBA-1 protein in microglial cells was also determined by western blots.

### Western Blotting and MAPK Determination

Phosphorylation of ERK1/2 was determined by western blots in microglial cells stimulated with PKall and NCD for 10 min. Following this microglial cells were lysed using RIPA buffer supplemented with protease inhibitor (1 mM PMSF, 1 mM Benzamidine, 2 μg/ml Aprotinin, 10 mM Sodium Fluoride, 2 mM Sodium Orthovanadate, 1 mM Sodium Pyrophosphate, and 2 μg/ml Leupeptin). The microglial cell lysate was then centrifuged, and the protein concentration of the supernatant was quantified by Lowry assay Kit (Bio-Rad, United States). Equal amounts of protein samples were subjected to 10% SDS-PAGE analysis and the gel containing the resolved proteins was then transferred onto nitrocellulose membranes. The membranes were then blocked with 3% BSA (Sigma-Aldrich, United States) for 1 h, followed by blotting with primary rabbit monoclonal anti-anti-phospho-P44/42 MAPK (ERK 1/2) antibodies (T202/Y204), 4370S), and rabbit monoclonal anti-total ERK 1/2 (T-44/42, 137F5, 4695S) antibodies, at 4°C overnight, followed by incubation with HRP-linked anti-rabbit antibody at room temperature for 1 h. All antibodies were purchased from Cell Signaling Technology (Danvers, MA, United States). Protein signals were detected using an ECL Detect Kit and visualized using Chemidoc MP imaging system (Bio-Rad, Hercules, CA). Image J software (NIH, United States) was utilized to quantify the bands and the ratio of phosphorylated ERK1/2 relative to total ERK1/2 was acquired and compared to control untreated microglial cells.

### Systems Biology and Subnetwork Enrichment Interactome Analysis

For the interactome analysis, the Elsevier’s Pathway Studio version 10.0 (https://www.elsevier.com/solutions/pathway-studio-biological-research) was used to establish the relationships among the different validated proteins relevant to microglial-PKall interaction using the ResNet database. Proteome interactome network was generated using “direct interaction” algorithm to map cellular processes and interactions among the genes of the altered proteins. For the brain and circulation deduced interactions, “Subnetwork Enrichment Analysis” (SNEA) algorithm was selected to extract statistically significant altered functional pathways pertaining to the validated proteins. SNEA utilizes Fisher’s statistical test to determine if there are nonrandom associations between two categorical variables organized by specific relationships. SNEA starts by creating a central “seed” from all relevant entities in the database and retrieving associated entities based on their relationship with the seed (that is, binding partners, expression targets, protein modification targets, regulation targets).

### Statistical Analysis

Descriptive analysis was conducted on each group and summary statistics were reported in terms of mean ± SE. Normality of the data was assessed using the Shapiro-Wilk test for normality. Comparison between the different groups was conducted using the nonparametric Kruskal-Wallis test and Bonferroni adjusted *p*-values were reported to account for multiple comparisons. In addition, Mann-Whitney U test was conducted when comparison was done between two groups only. Significance was considered at *p* < 0.05 and statistical analyses were conducted using the SPSS (Statistical Package for the Social Sciences) software.

## Results

### Basal Expression of Components of the Plasma Kallikrein-Kinin System in Microglial Cells

**FIGURE 1:**
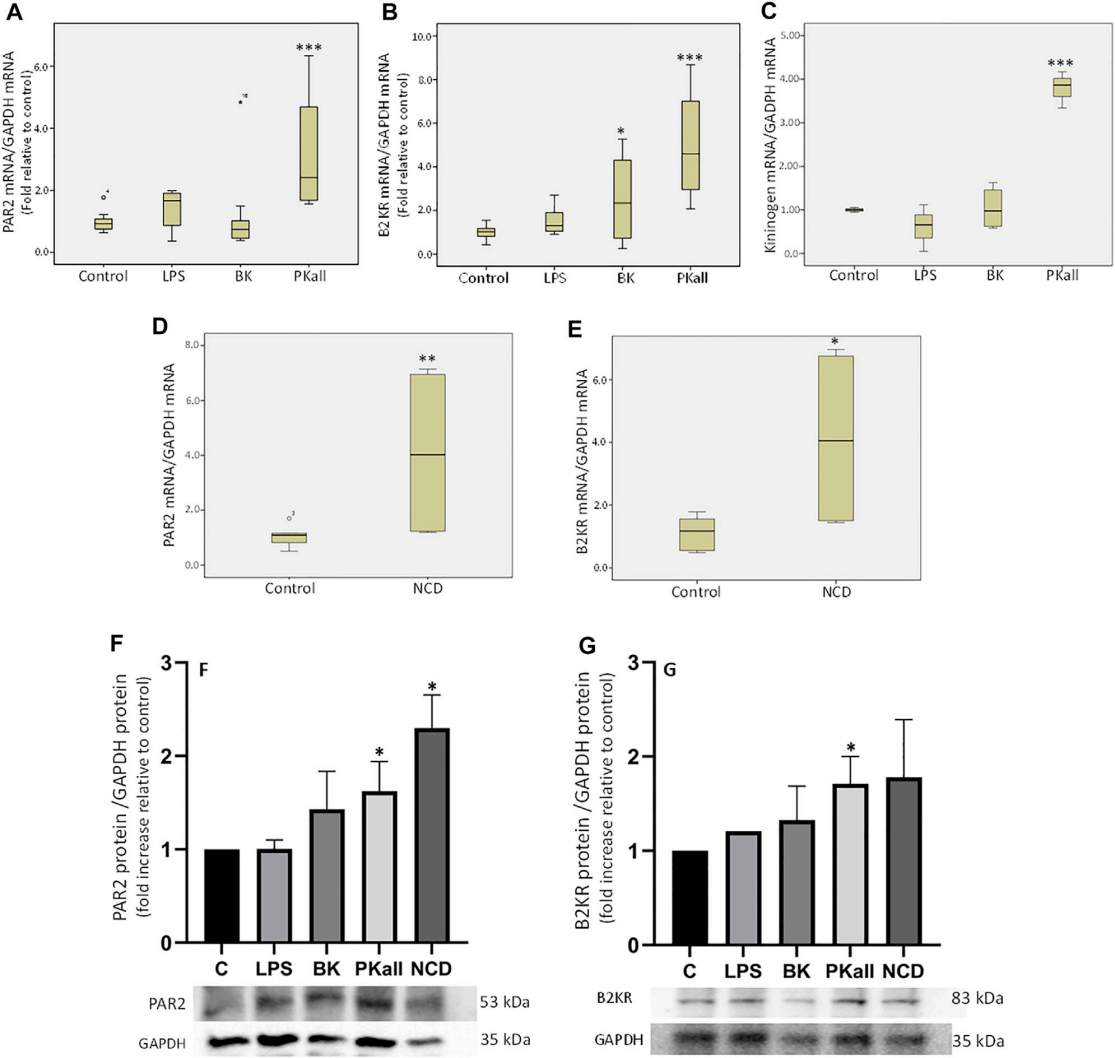
Expression of components of the plasma kallikrein-kinin system (PKKS) in microglial cells. Microglial cells grown in 2% FBS were stimulated with LPS (100 ng/ml), BK (0.1 µM), PKall (2.5 ng/ml) and NCD (16.5 µg protein/ml) for 24 h. The boxplots demonstrate the mRNA levels of **(A)** PAR 2, **(B)** B2KR and **(C)** KNG expressed relative to GAPDH mRNA levels assessed concomitantly at the same time in the same samples (*p ≤ 0.05, ***p ≤ 0.005 vs. Control, *n* = 5–7). The boxplots **(D)** PAR 2 and **(E)** B_2_KR, demonstrate the mRNA levels of these receptors expressed relative to GAPDH mRNA levels assessed concomitantly at the same time in the same samples in response to neuronal cell debris (*p ≤ 0.04, **p ≤ 0.033 vs. Control, *n* = 5). The bar graphs **(F)** and **(G)** represent the protein expression of PAR2 and B_2_KR relative to GAPDH protein levels measured concomitantly at the same time in the same samples in response to LPS, BK, PKall, and NCD (*p ≤ 0.04 vs. C, *n* = 4).

The first series of experiments were designed to examine whether microglial cells express components of the PKKS and to identify the factors that can modulate their expression. The data shown in Figure 1A demonstrates that in microglial cells, PKall significantly increases the gene expression of PAR 2 by 3-fold (3.18 ± 0.24, *n* = 5,***p ≤ 0.005), compared to untreated control cells (1.00 ± 0.03, *n* = 7). LPS, which is a reference for microglia activation, had no significant effect on the level of expression of PAR 2 compared to control cells (1.36 ± 0.32 vs. 1.00 ± 0.15, LPS vs. Control, respectively, *p* = 0.127). BK treatment also had no significant effect on PAR 2 gene expression compared to untreated cells (1.22 ± 0.47 vs. 1.00 ± 0.15, BK vs. Control, respectively, *p* = 0.275). Data depicted in Figure 1B indicates that the gene expression of B_2_KR was increased significantly by 2.4-fold in response to BK (2.41 ± 0.62 vs. 1.00 ± 0.14, BK vs. Control, respectively, *p ≤ 0.05) and by 5-fold in response to PKall treatment (4.99 ± 1.40 vs. 1.00 ± 0.14, PKall vs. C, respectively, ***p ≤ 0.005).

LPS had no significant effect on B_2_KR gene expression in microglial cells (1.57 ± 0.33 vs. 1.00 ± 0.14, LPS vs. Control, respectively, *p* = 0.275, Figure 1B). With respect to *KNG*, PKall resulted in a 3.8-fold increase in the gene expression of *KNG* compared to control cells (3.79 ± 0.24 vs. 1.00 ± 0.03, PKall vs. C, respectively, ***p ≤ 0.001, Figure 1C). Treatment of microglial cells with LPS and/or BK did not significantly alter the mRNA levels of KNG compared to controls (0.61 ± 0.31, 1.04 ± 0.24 vs. 1.00 ± 0.03, LPS, BK vs. Control, respectively, *p* = 0.513, Figure 1C). These findings demonstrate for the first time that B_2_KR, PAR2, and KNG are expressed in microglia and their expression is modulated by PKall.

To determine whether NCD regulates the expression of PKKS components, microglia cells were exposed to neuronal cell debris for 24 h and the results are depicted in Figures 1D,E. Exposure of microglia cells to NCD resulted in a significant increase in the gene expression levels of PAR 2 (4.10 ± 1.65 vs. 1.07 ± 0.16, NCD, vs. control, respectively, **p ≤ 0.033, Figure 1D), and B_2_KR (4.13 ± 1.52 vs. 1.12 ± 0.22, NCD vs. control, respectively, *p ≤ 0.04, Figure 1E). No significant effect on the expression levels of *KNG* (0.61 ± 0.18 vs. 1.08 ± 0.21, NCD vs. control, respectively, and PKall (1.24 ± 0.44 vs. 1.01 ± 0.06, NCD vs. control, respectively) was observed. This finding provides the first demonstration that NCD can stimulate the expression B_2_KR and PAR2 in microglial cells.

The protein expression of B_2_KR and PAR2 were also examined in microglial cells in response to LPS, BK, PKall, and NCD and the results are shown in Figures 1F,G. Our findings indicated that LPS and BK treatment did not significantly increase the protein expression of PAR2 compared to unstimulated control cells [*p* < 0.43 and *p* < 0.435, LPS (*n* = 3) and BK (*n* = 3) vs. C (*n* = 4), respectively, Figure 1F]. Treatment of microglial cells with PKall and NCD, significantly increased the protein expression of PAR2 compared to control cells [*p* < 0.04, PKall (*n* = 4) and NCD (*n* = 4) vs. C (*n* = 4), Figure 1F]. This finding is the first to demonstrate that PKall and NCD can stimulate the expression of PAR2 in microglial cells. The protein expression of B_2_KR was not significantly increased in response to LPS and BK compared to controls [*p* < 0.435, LPS (*n* = 3) and BK (*n* = 3) vs. C (*n* = 4), respectively, Figure 1G]. PKall treatment significantly increased the protein expression of B_2_KR compared to control [*p* < 0.04 (*n* = 4) vs. C (*n* = 4), Figure 1G]. NCD stimulated the protein expression of B2KR by 1.78 fold compared to control, but this increase was not significant [*p* < 0.608, NCD (*n* = 4) vs. C (*n* = 4), Figure 1G].

### Microglial Cell Activation

**FIGURE 2:**
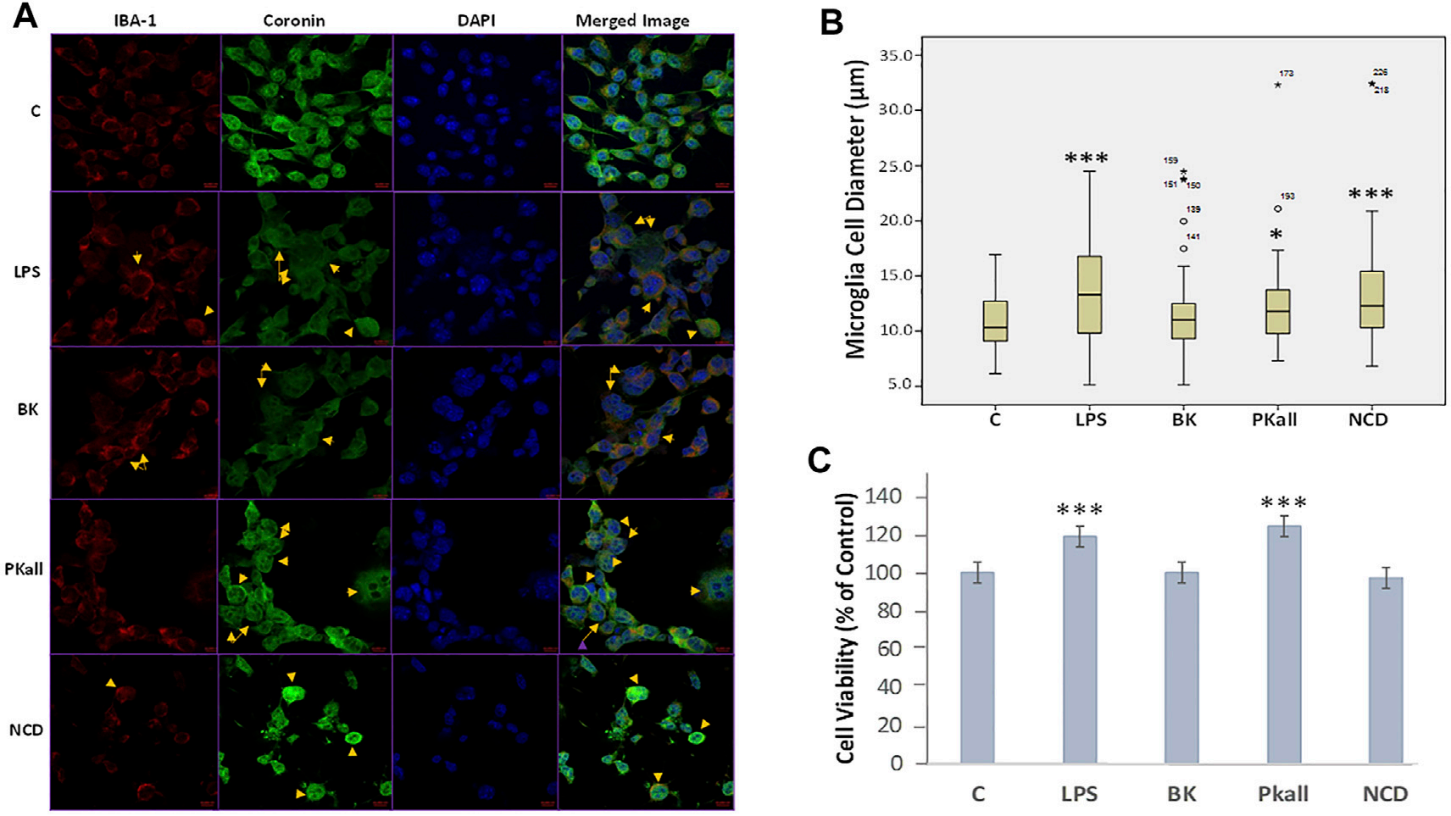
Immunofluorescence and morphological changes of activated microglial cells. N9 microglial cells cultured on coverslips were stimulated with vehicle control (c), LPS (100 ng/ml), BK (0.1 µM), PKall (2.5 ng/ml), and NCD (16.5 µg protein/ml) for 24 h **(A)** Immunostaining was conducted using a specific anti-IBA-1 antibody (red), anti-Coronin antibody (green), DAPI to recognize the nucleus (blue), and the merged image. The yellow arrows indicate a change in the morphology of the microglial cells adopting a more rounded amoeboid shape indicative of phagocytosis. The immunofluorescence intensity of IBA-1 and coronin was not different between the groups. **(B)** Boxplot demonstrating the changes in microglial cell diameter (µm) in response to LPS, PKall, BK. and NCD stimulation measured by confocal microscopy. Data are representative of at least 50 cells from different representative images and expressed as mean ± SE (*p ≤ 0.03, ***p ≤ 0.001 vs C). **(C)** Cell viability of microglial cells. Microglial cell viability was determined by MTT assay in response to LPS, BK, PKall, and NCD. The bar graph represents the microglial cell viability expressed as a percentage of control (***p ≤ 0.001, LPS or PKall vs. C, respectively, *n* = 12).

We next sought to determine whether microglial cells would be activated in response to LPS, PKall, BK, and NCD treatment, by evaluating changes in their morphology as well as the immunofluorescence staining intensity of IBA-1 (red), Coronin (green), and Dapi (blue) by confocal microscopy. IBA-1 is a macrophage-specific calcium-binding protein that is involved in phagocytosis in activated microglia and Coronin is an actin-binding protein that is associated with phagocytosis in macrophages (Ahmed et al., 2007; Das et al., 2020). The results shown in Figure 2A *,* reveal that the immunofluorescence of IBA-1 and Coronin staining in microglial cells exposed to LPS, PKall, BK, and NCD compared to control unstimulated cells (Figure 2A). Detection of coronin and IBA-1 protein in microglial cells in response to LPS, BK, PKall, and NCD was also determined by western blots and the results are shown in Supplementary Figure S1.

The changes in microglial cell morphology, which is indicative of microglial cell activation and phagocytosis in response to LPS, PKall, BK, and NCD stimulation were examined by confocal microscopy. The results shown in Figure 2B, demonstrated that LPS, PKall, and NCD significantly increased microglial cell diameter by more than 10% compared to control unstimulated cells (***LPS = 19.23 ± 0.68 µm, *PKall = 17.58 ± 0.76 µm, ***NCD = 19.10 ± 1.18 µm vs. C = 15.99 ± 0.33 µm, *p ≤ 0.03, ***p ≤ 0.001 vs. control, respectively). BK had no significant effect on microglial cell diameter compared to unstimulated control cells (BK = 16.67 ± 0.53 µm vs. C = 15.99 ± 0.33 µm, *p* = 0.616, Figure 2B *.*

### Cell Viability of Microglial Cells

Microglial cell viability was examined in response to LPS, BK, PKall, and NCD by the MTT assay. The results depicted in Figure 2C, demonstrated an absence of toxicity with LPS and PKall treatment resulting in a significant increase in cell viability compared to the unstimulated cells (***p ≤ 0.001, LPS or PKall vs. C, respectively). Neither BK nor NCD had any significant effect on microglial cell viability or toxicity compared to control unstimulated cells (Figure 2C).

### Activation of Inflammatory Signals in Microglia

**FIGURE 3:**
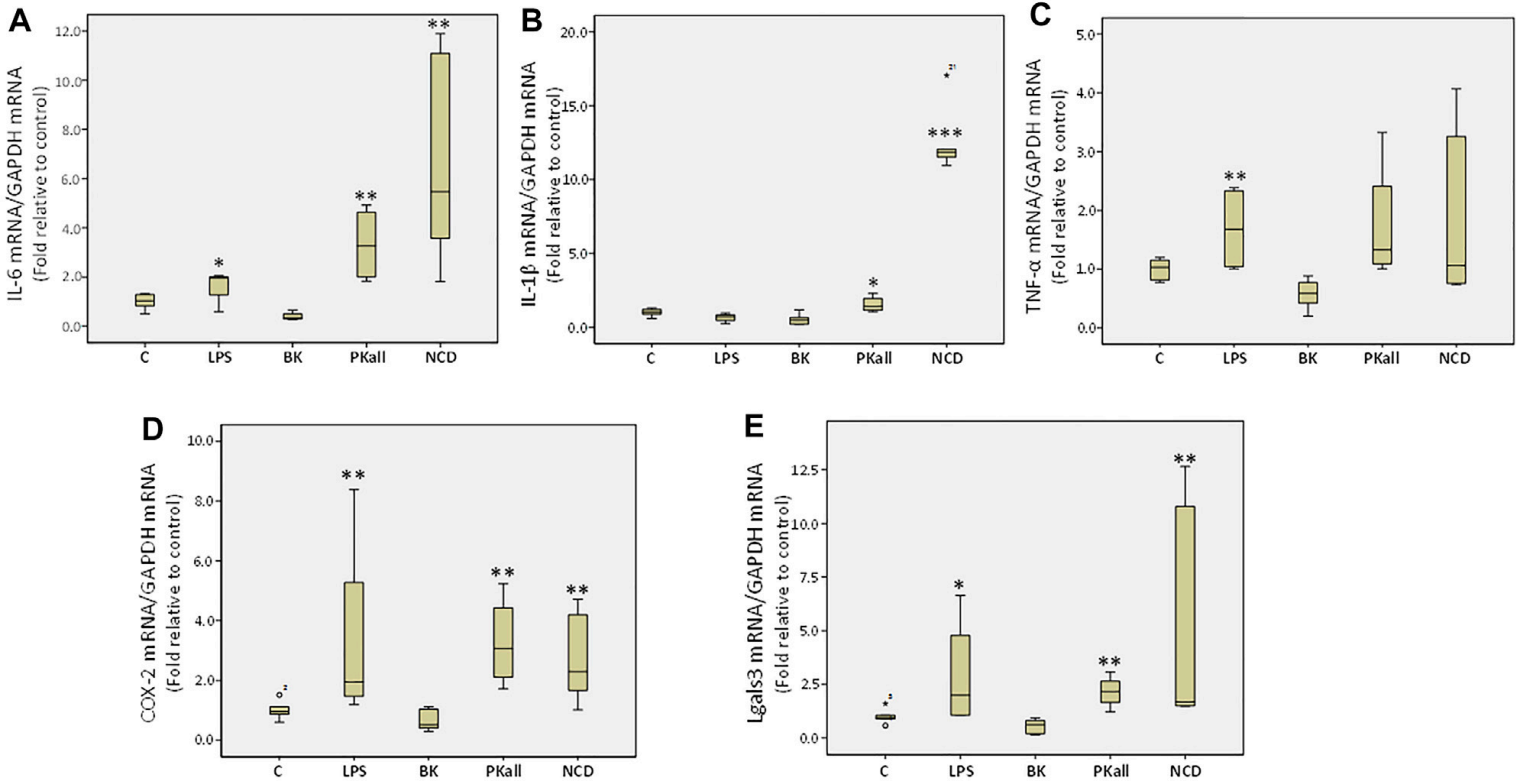
Expression of inflammatory mediators in microglial cells. The gene expression levels of inflammatory cytokines measured in N9 microglial cells exposed to LPS (100 ng/ml), BK (0.1 µM), PKall (2.5 ng/ml) and NCD (16.5 µg protein/ml) for 24 h. Boxplot graphs depicts the changes in mRNA levels of **(A)** interleukin-6 (IL-6), **(B)** interleukin-1 β (IL-1β), (**C)** tumor necrosis factor-alpha (TNF-α), **(D)** cyclooxygenase-2 (COX-2), and **(E)** galectin-3 (Lgals3) expressed relative to control mRNA levels and to GAPDH mRNA levels were determined in the same samples at the same experiment (*p ≤ 0.05,**p ≤ 0.01, ***p ≤ 0.004 vs. Control, *n* = 5).

To further evaluate the capacity of microglia to upregulate pro-inflammatory signals upon activation, we determined the levels of pro-inflammatory factors secreted by microglial cells in response to LPS, BK, PKall, and NCD treatment. We assessed the neo-expression of inflammatory cytokines such as interleukins 6 and 1-beta (IL-6, IL-1β) and tumor necrosis factor-alpha (TNF-α) as well as the expression of cyclooxygenase-2 (COX-2) and galectin-3 (Lgals3) whose upregulation is linked to neuro-inflammatory conditions, distinctive of microglial activation and augmentation of inflammatory mediators (Vijitruth et al., 2006; Siew et al., 2019). Microglial cells were exposed to LPS, BK, PKall, and NCD for 24 h. The fold change in the gene expression levels of IL-6, IL-1β, TNF-α, COX-2, and Lgals3 expressed relative to control are shown in Figures 3A–E *.* The gene expression of IL-6 (Figure 3A) was markedly enhanced in response to LPS (1.64 ± 0.35, *p ≤ 0.05), PKall (3.32 ± 0.77, and NCD 6.77 ± 2.01, **p ≤ 0.01) compared to control cells (1.00 ± 0.13). BK had no significant effect on IL-6 gene expression. PKall and NCD also significantly increased the gene expression levels of IL-1β compared to controls (*PKall = 1.54 ± 0.28, ***NCD = 12.67 ± 1.11 vs. 1.00 ± 0.15, *p ≤ 0.05, ***p ≤ 0.004 vs. Control, respectively, Figure 3B). LPS and BK had no significant effect on IL-1β gene expression.

LPS significantly increased TNF-α expression compared to control (LPS = 1.68 ± 0.37 vs. control = 1.00 ± 0.07, **p ≤ 0.028, Figure 3C). Exposure of microglial cells to BK, PKall, and NCD had no significant effect on the expression levels of TNF-α (Figure 3C). The gene expression of COX-2 (Figure 3D) was increased significantly by about 3.3-fold in response to LPS (3.37 ± 1.68), 3.27-fold in response to PKall (3.27 ± 0.76) and about 2.78-fold in response to NCD (2.78 ± 0.72), **p ≤ 0.01, compared to control cells (1.00 ± 0.12). Moreover, the gene expression levels of Lgals3 were also markedly enhanced in response to LPS (*p ≤ 0.05), PKall (**p ≤ 0.01), and NCD (**p ≤ 0.01), compared to control cells (Figure 3E). BK had no significant effect on the gene expression levels of COX-2 and Lgals3 (Figure 3D,E).These findings provide the first evidence that PKall can promote the expression of pro-inflammatory factors, a key feature of microglial cell activation.

**FIGURE 4:**
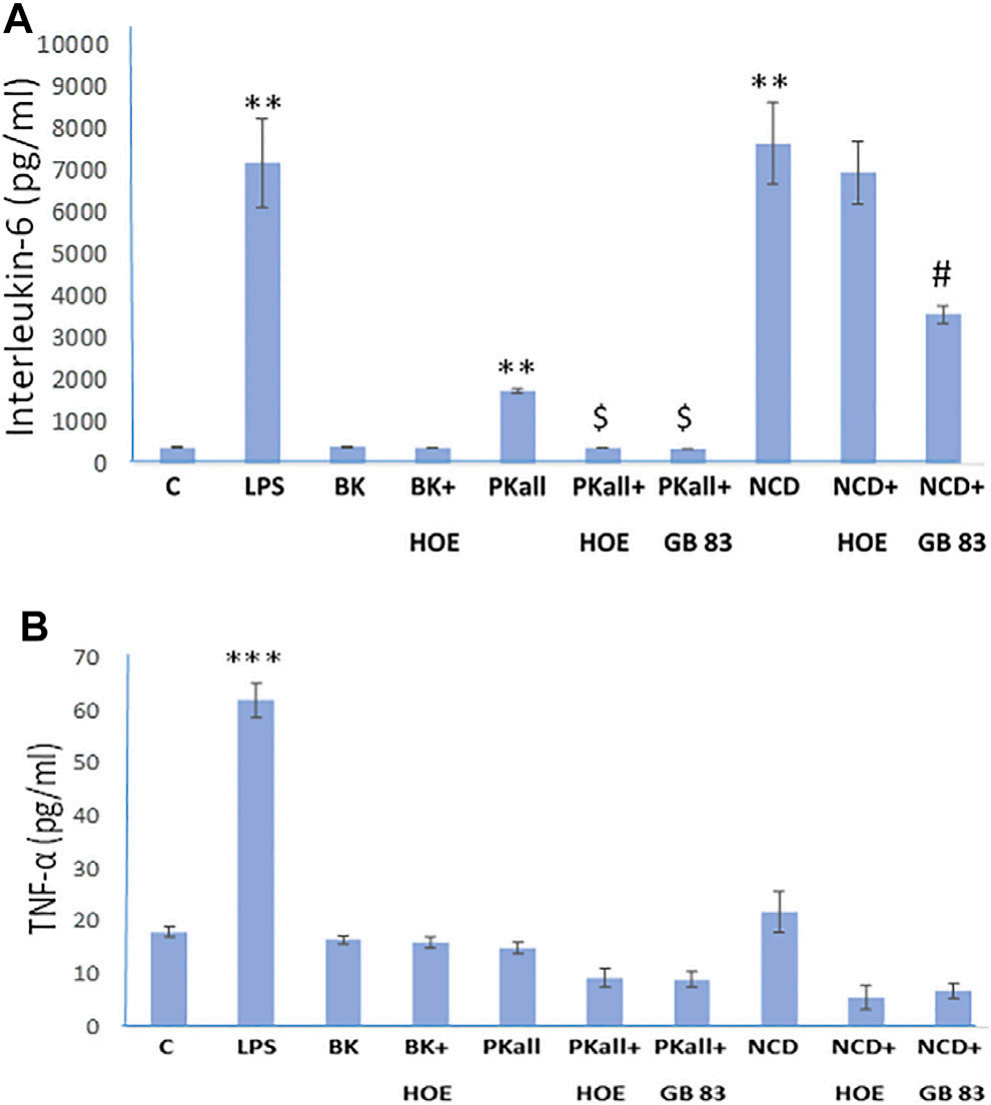
Engagement of PAR 2 and B2KR in cytokine production in the microglial cell. Microglial cells were stimulated for 24 h with PKall (2.5 ng/ml) or NCD (16.5 µg protein/ml) in the presence and absence of a B_2_KR antagonist HOE 140 (1 µM) and a PAR 2 antagonist GB 83 (2 μM). Bar graphs depicts the levels of **(A)** interleukin-6 (IL-6, pg/ml), and **(B)** tumor necrosis factor-alpha (TNF-α, pg/ml) measured in the supernatant media by ELISA (**p ≤ 0.032, ***p ≤ 0.008 vs. C; $*p* ≤ 0.008, PKall vs. PKall + HOE 140, or PKall + GB 83; #*p* ≤ 0.025, NCD vs. NCD + GB 83, *n* = 5).

We next sought to define the pathway through which PKall and NCD promote microglial cell activation and to identify the receptors through which this process occurs. Our data demonstrated that both PKall and NCD stimulate the expression of B_2_KR and PAR 2 in microglial cells. Hence we speculated that PKall and NCD share a common mechanism to enhance pro-inflammatory signals by engaging B_2_KR and PAR 2. To address this notion, microglial cells were stimulated for 24 h with PKall and NCD in the presence and absence of a B_2_KR antagonist HOE 140 (0.1 μM) or PAR 2 antagonist GB 83 (2 μM). The levels of IL-6 and TNF-α are depicted in Figures 4A,B. LPS, markedly increased the levels of IL-6 compared to unstimulated cells (7,181 ± 1,054 pg/ml vs. 406 ± 12 pg/ml, LPS vs. C, **p ≤ 0.032, *n* = 5, Figure 4A. Exposure of microglia to PKall significantly enhanced the production of IL-6 levels compared to control (1,756 ± 45 pg/ml vs. 406 ± 12 pg/ml, **PKall vs. C, *p* ≤ 0.032, *n* = 5). This increase in IL-6 levels produced by PKall was completely inhibited in the presence of HOE 140 or GB 83 (1756 ± 45 pg/ml vs. 400 ± 4 pg/ml, 375 ± 4 pg/ml, PKall vs. PKall + HOE 140, PKall vs. PKall + GB 83, $*p* ≤ 0.008, respectively, Figure 4A). Exposure of microglia to NCD significantly increased the levels of IL-6 compared to control cells (7,649 ± 976 pg/ml vs. 406 ± 12 pg/ml, **p ≤ 0.032, Figure 4A). The increase in IL-6 levels observed in response to NCD was significantly reduced by about 50% in the presence of GB 83 (7,649 ± 976 pg/ml vs. 3,575 ± 209 pg/ml, NCD vs NCD + GB 83, #*p* ≤ 0.025, Figure 4A). However, the B_2_KR antagonist did not significantly influence the increase in IL-6 levels induced by NCD (7,649 ± 976 pg/ml vs. 6,955 ± 743 pg/ml, NCD vs. NCD + HOE 140, *p* = 0.602). BK treatment of microglial cells did not significantly affect IL-6 levels compared to unstimulated control cells (420 ± 12 pg/ml vs. 406 ± 12 pg/ml, BK vs. Control, respectively, *p* = 0.347, Figure 4A). These findings provide the first evidence that PKall and NCD stimulate the production of IL-6 levels in microglial cells via engagement of PAR 2.

Exposure of microglia to LPS resulted in a 3-fold increase in the levels of TNF-α compared to control cells (62 ± 3 pg/ml vs. 18.1 ± 0.9 pg/ml, LPS vs. Control, respectively, ***p ≤ 0.008, Figure 4B). On the other hand, treatment of microglial cells with BK, PKall, and/or NCD had no significant effect on TNF-α protein levels (Figure 4B).

### Autophagy and Microglial Inflammatory Signals

**FIGURE 5:**
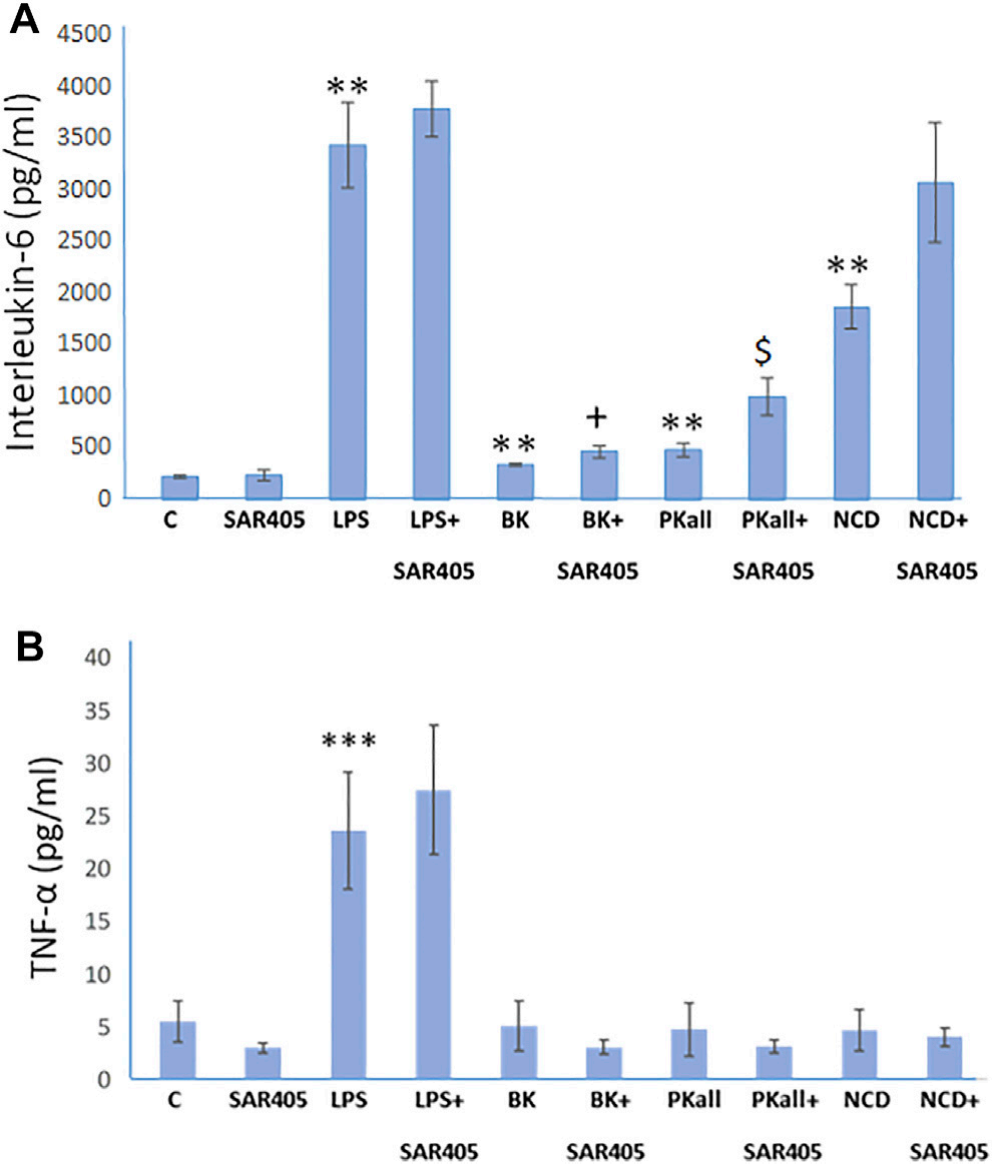
Autophagy and microglial inflammatory signals. To determine if autophagy plays a role in the microglial inflammatory response, microglial cells were exposed to LPS (100 ng), BK (10^−7^ M), PKall (2.5 ng/ml), or NCD (16.5 µg protein/ml) for 24 h, in the presence and absence of SAR 405 (1 µM), a phosphatidylinositol 3-kinase, catalytic subunit type 3 (PIK3C3) inhibitor, that inhibits autophagy. The bar graph shows the levels of **(A)** interleukin-6 (IL-6, pg/ml) and **(B)** tumor necrosis factor-alpha (TNF-α, pg/ml) released into the media and measured by ELISA (**p ≤ 0.02 or less vs. C, ***p ≤ 0.004 vs. C; $*p* ≤ 0.009, PKall vs. PKall + SAR405; +*p* ≤ 0.037, BK vs. BK + SAR405, *n* = 6).

The process of autophagy has been implicated in microglial function and its dysregulation, has been shown to influence innate immune roles that include inflammation and phagocytosis (Plaza-Zabala et al., 2017; Jin et al., 2018). In order to determine if autophagy plays a role in the microglial inflammatory response to LPS, BK, PKall, and NCD, we examined whether inhibition of autophagy will accentuate markers of pro-inflammatory cytokines such as IL-6 and TNF-α. SAR 405 (1 µM), a phosphatidylinositol 3-kinase, catalytic subunit type 3 (PIK3C3) inhibitor, that inhibits the initial steps of macroautophagy (Pasquier, 2015) was used in the current study. We have previously shown that SAR405 prevented the lipidation of LC3 (from LC3-I, un-lipidated to LC3-II, lipidated) in two macrophage cell models, bone marrow derived macrophages and HELA cells (Supplementary Figure S2). Microglial cells were exposed to LPS, BK, PKall, and NCD for 24 h, in the absence or presence of 1 µM SAR 405. Levels of IL-6 and TNF-α released into the media are depicted in Figure 5A,B. Treatment of microglial cells with SAR 405 alone had no significant effect on the basal levels of IL-6 (239 ± 54 pg/ml vs. 221 ± 14 pg/ml, SAR 405 vs. Control, respectively, *p* = 0.873, *n* = 6, Figure 5A). Exposure of microglial cells to LPS produced a 15-fold increase in IL-6 levels compared to basal control levels (LPS = 3,423 ± 410 pg/ml vs. Control = 221 ± 14 pg/ml, **p ≤ 0.02). In the presence of SAR 405, LPS treatment increased the IL-6 levels from 3,423 ± 410 pg/ml to 3,770 ± 269 pg/ml, but this increase was not significant (*p* = 0.465). On the other hand, PKall produced a 2.2-fold increase in the levels of IL-6 compared to unstimulated control cells (482 ± 61 pg/ml vs. 221 ± 14 pg/ml, PKall vs. Control, respectively, **p ≤ 0.01, Figure 5A). However, in the presence of SAR 405, PKall treatment significantly increased the IL-6 levels from 482 ± 61 pg/ml to 996 ± 176 pg/ml, $*p* ≤ 0.009, Figure 5A).

Along the same lines, NCD also resulted in 8-fold significant increase in IL-6 levels compared to control cells (1858 ± 214 pg/ml vs 221 ± 14 pg/ml, NCD vs. Control, **p ≤ 0.01, Figure 5A). Moreover, in the presence of SAR 405, exposure of microglial cells to NCD resulted in a further increase in IL-6 levels by 1.7-fold, which is 14-fold higher than basal control levels. BK treatment resulted in a significant increase in IL-6 levels, **p ≤ 0.01. Moreover, when comparing BK to BK + SAR 405 inhibitor there was a significant increase in IL6 levels with +*p* ≤ 0.037, Figure 5A.

With respect to TNF-α levels, LPS as shown previously, resulted in a significant increase in TNF-α levels (***p ≤ 0.001) compared to control unstimulated cells (Figure 5B). In the presence of SAR 405, LPS did not significantly increase TNF-α levels. Treatment of microglial cells with BK, PKall, and/or neuronal cell debris had no significant effect on TNF-α levels compared to basal levels and this effect was not significantly modified in the presence of SAR 405 (Figure 5B). These findings provide the first evidence of a link between PKall and autophagy in the modulation of the inflammatory response in microglial cells.

### Role of Extracellular Regulated Kinase ½ in IL-6 Production in Microglial Cells

**FIGURE 6:**
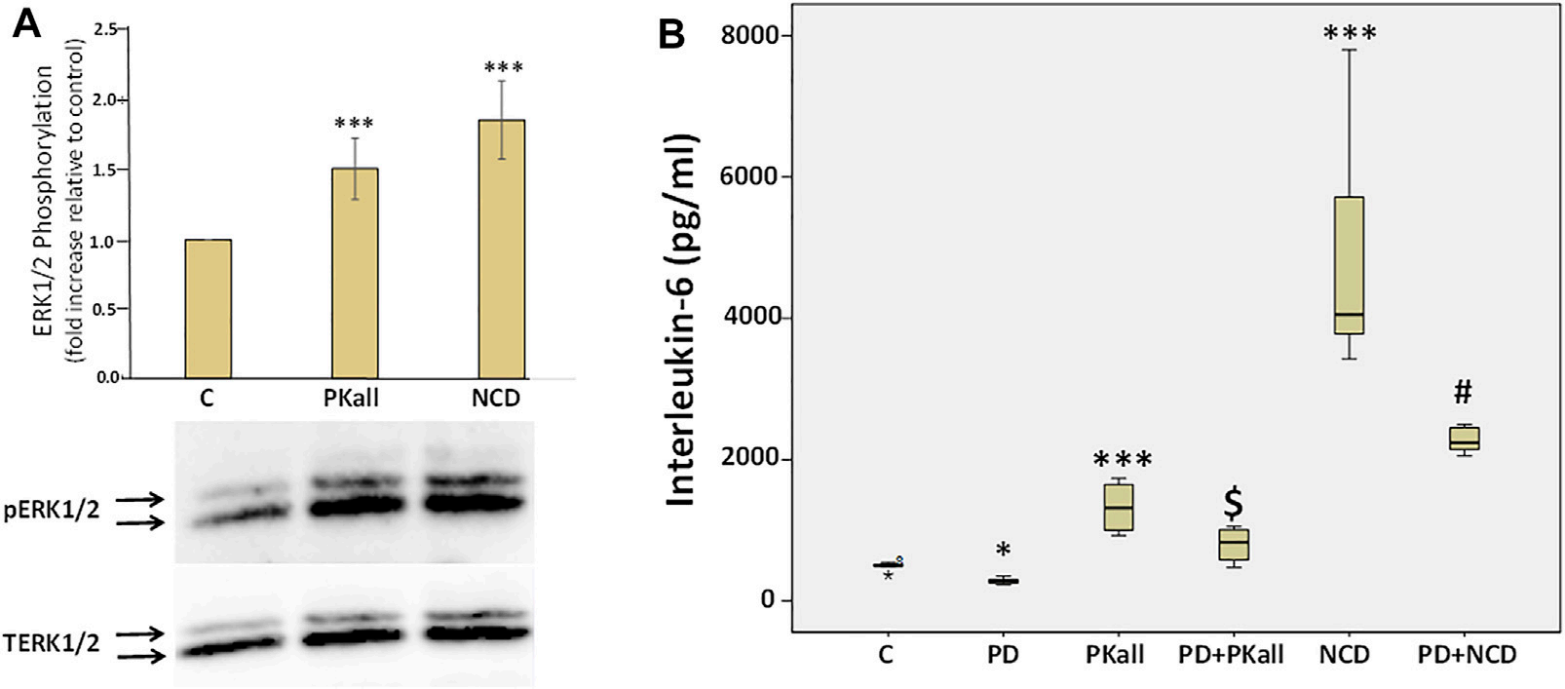
Role of extra cellular regulated kinase1/2 (ERK1/2) in PKall and neuronal cell debris induced IL-6 production in microglial cells. **(A)** PKall and NCD stimulated ERK 1/2 phosphorylation (pERK 1/2) in microglial cells. N9 microglial cells were exposed to PKall (2.5 ng/ml) and/or NCD (16.5 μg protein/ml) for 10 min pERK and total ERK (TERK) levels were assessed by western blot. Bar graph represents the fold change in pERK relative to TERK protein levels (*p < 0.001 *n* = 6). **(B)** N9 microglial cells were stimulated with PKall (2.5 ng/ml) and/or NCD 16.5 μg protein/ml) for 24 h in the presence and absence of the MEK 1 inhibitor PD98059 (25 μM). Box plot represents the production and release of IL-6 levels into the media measured by ELISA in the different treatment groups. (*p ≤ 0.05, control vs. PD; ***p-value ≤ 0.001, PKall or NCD vs. Control; $*p* ≤ 0.004, PKall vs. PD + PKall; #*p* ≤ 0.002 NCD vs. PD + NCD, *n* = 6).

To explore potential mechanisms through which PKall and NCD promote microglial cell activation and inflammatory cytokine production, we first examined if PKall and NCD will activate the MAPK pathway and whether inhibition of MAPK will modulate the levels of cytokines produced in response to PKall and NCD. Figure 6 A depicts the effects of PKall and NCD on ERK1/2 phosphorylation, pERK1/2 (indicative of ERK1/2 activation) in microglial cells. Total ERK1/2 (TERK1/2) levels were also assessed by western blot in the same samples and represented the protein control for equal protein loading. The activation of ERK1/2 was determined from the ratio of pERK1/2 relative to TERK1/2 and the bar graph represents the fold change in pERK relative to TERK protein levels. Exposure of microglial cells to PKall and/or NCD for 10 min induced the phosphorylation of ERK 1/2 compared to unstimulated control cells (***p ≤ 0.002, Figure 6A).

We subsequently sought to determine if ERK1/2 contributes to the production of IL-6 levels in response to PKall and/or NCD treatment. Microglial cells cultured in 12-well plates were treated with PKall and NCD in the absence and presence of MEK (MAP kinase) inhibitor (PD-98059, 25 μM, Cayman Co., Ann Arbor, MI, United States) for 24 h. PD98059 is a specific inhibitor of MEK 1 (MAP kinase), an upstream activator of ERK 1/2. PD98059 interacts with MEK1 in its inactive state and hence prevents its phosphorylation by the upstream activator c-Raf. When deactivated, MEK1 is incapable of phosphorylating ERK 1/2, its downstream target.

The data depicted in Figure 6B demonstrate that both PKall and NCD produced a significant increase in the levels of IL-6 compared to unstimulated cells. However, this increase in the production of IL-6 levels in response to PKall and NCD was significantly decreased in the presence of the MEK1 inhibitor. These results point to a mechanistic role for ERK 1/2 in IL-6 production in response to PKall and NCD in microglial cells.

### Interactome Networks of Altered Genes

**FIGURE 7:**
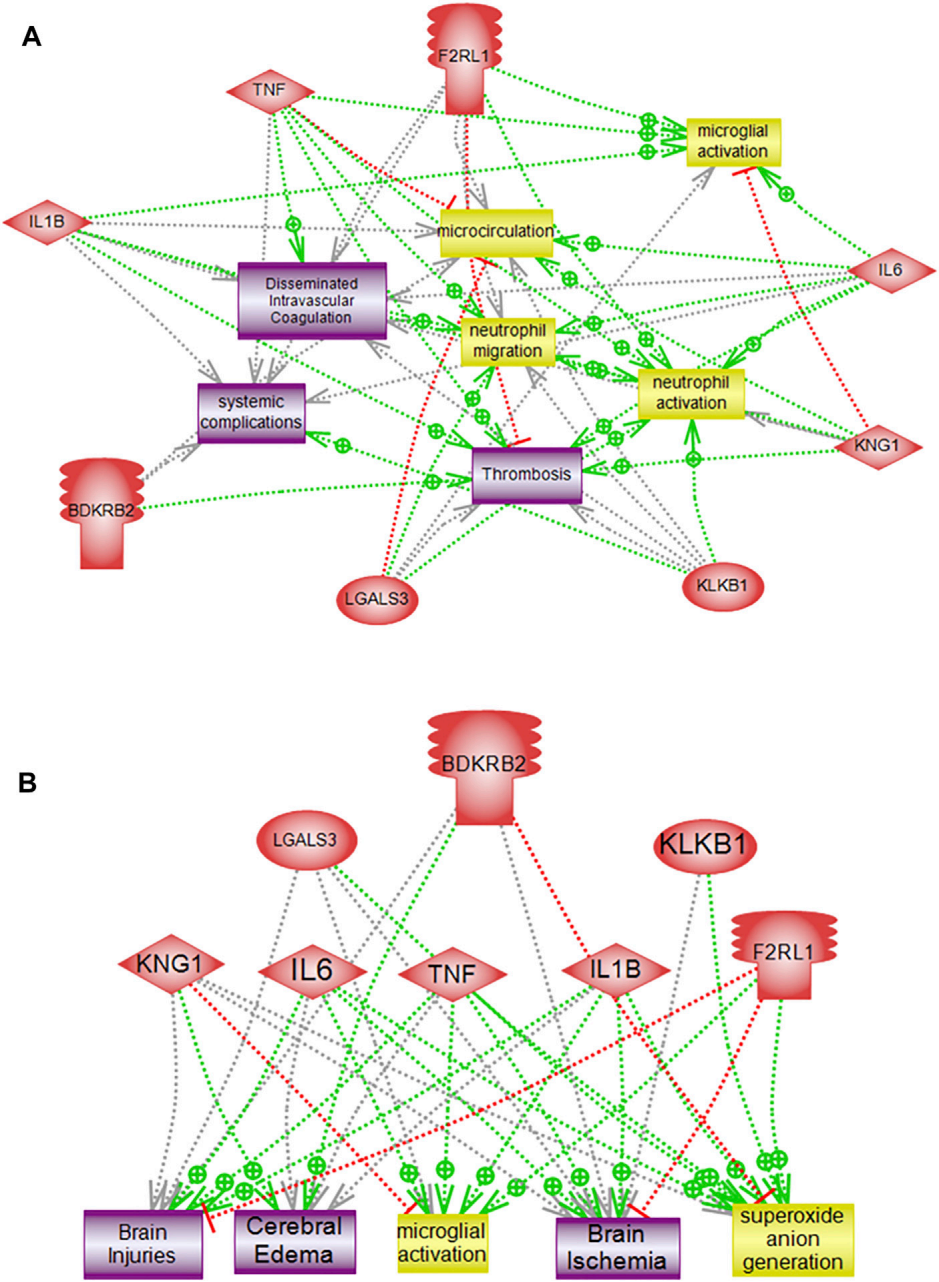
Pathway analysis of altered genes demonstrating direct functional interactions interactome networks in microglial cells. **(A)** Systemic related interactome, **(B)** Brain-related interactome. F2RL1 (Protease-activated receptor 2), IL-6 (Interleukin-6), KNG1 (Kininogen), KLKB1 (PKall), LGALS3 (Galectin-3), B_2_KR (Bradykinin 2 receptor), IL-1B (Interleukin-1β), TNF-α (Tumor necrosis factor-α), COX2 (cyclooxygenase2), LPS (Lipopolysaccharide).

Interactome mapping analysis were conducted to correlate altered protein changes with microglial activation centered on disease processes in order to help recognize functional elements or biological pathways linked to disease. Network analysis of the genes altered in response to PKall, NCD, BK, and LPS revealed highly linked direct interaction interactomes indicative of functional effects related to brain pathways as well as to systemic related pathways. The brain-related pathways demonstrated that PKall, KNG, B_2_KR, IL-6, TNF-α, IL-1β, LGALS-3, and PAR 2 were linked to superoxide anion generation and brain ischemia. Cerebral edema and brain injuries were associated with KNG, B_2_KR, IL-6, TNF-α, IL-1β, and LGALS-3, whereas microglial activation was linked to IL-6, TNF-α, IL-1β, LGALS-3, and PAR 2. Moreover, the systemic-related pathways revealed that systemic complications, microcirculation, thrombosis, neutrophil activation, neutrophil migration, and disseminated intravascular coagulation were linked to PKall, KNG, IL-6, TNF-α, IL-1β, and PAR 2 (Figures 7A,B). The interactome relationships, along with the references used to identify these results are shown in supplemental data).

## Discussion

Microglial cells are the main tissue occupants of the CNS that are vigorously engaged in the protection and upkeep of the hemostatic environment of the brain by modulating neuronal circuit plasticity and synaptic remodeling (Wang et al., 2015; Sousa et al., 2017; Stevens and Schafer, 2018). Microglia represent the chief drivers of the innate immunity in the brain, and, once faced with a challenge, they respond by changing their morphology and adopting a more phagocytic phenotype with a concomitant increase in the expression profiles and release of inflammatory cytokines and neurotransmitters (Salter and Stevens, 2017). The factors that promote microglial activation are not yet entirely defined, nor do we fully appreciate the pathways through which this activation occurs. In the current work, we have shown that microglial cells express B_2_KR, PAR 2, and *KNG* and their expression is enhanced in response to PKall stimulation. In addition, our data provide the first demonstration that neuronal cell debris can stimulate the expression of B_2_KR and PAR 2 in microglial cells. Furthermore, PKall and neuronal cell debris modulated microglial cell morphology and accentuated the expression of pro-inflammatory factors, a key event of microglial cell activation. Finally, the production of IL-6 in response to PKall and neuronal cell debris in microglial cells was mediated via engagement of PAR2, involvement of autophagy, and activation of the MAPK pathway. These data were substantiated via the bioinformatics analysis demonstrating the role of PKall and associated proteins in systematic and brain inflammatory processes.

The role and contribution of PKall to aging and neurodegenerative disorders are still being unraveled (Albert-Weissenberger et al., 2014; Nokkari et al., 2018; Strickland 2018). Higher levels of circulating PKall activity were associated with augmented *KNG* cleavage resulting in the greater generation of BK levels which could influence vascular dysregulation in states of neurodegenerative diseases (Zamolodchikov et al., 2015; Simoes et al., 2019; Park et al., 2021). Mice lacking the PKall gene (klkb1), displayed reduced brain infarctions and fewer neurological shortfalls compared to wild-type mice when subjected to brief central cerebral artery obstruction (Gob et al., 2015). Given the critical role microglial cell activation plays in neurodegenerative diseases, our studies may provide potential mechanistic pathways linking PKall to neuronal injury. In this regard, the expression of PKall, *KNG*, B_2_KR, and PAR 2 in microglial cells implicate a potential role for these factors in modulating microglial cell function and activation. Our findings demonstrated that PKall significantly increased microglial cell expression of inflammatory mediators such as IL-6, IL-1β, COX-2, and Lgals-3 that are known to play critical roles in microglial cell dysregulation. The cytokine IL-6 is one of the main regulators of microglial activation that functions as a central modulator of inflammatory and innate immune responses in the CNS (Erta et al., 2012). IL-6 levels increase markedly in response to acute injury such as TBI and ischemia and chronic neurodegenerative disorders (Boche andNicoll, 2013; Yang et al., 2013). Interactions between IL-6 and other cytokines such as IL-1β promote substantial inflammatory responses in the microglia within the CNS environment to initiate the process of neuronal apoptosis (Wang et al., 2005; Neher and Cunningham, 2019; Recasens et al., 2021). Induction of COX-2 has been shown to play a pivotal role in the activation of microglial cells and the development of inflammatory immune response (Guan and Wang, 2018). Galectin-3 has been shown to regulate microglial activity and is essential for the activation and proliferation of microglial cells as a result of ischemic injury (Boza-Serrano et al., 2014; Siew et al., 2019). Furthermore, our findings also demonstrated that exposure of microglial cells to neuronal cell debris induced the expression of inflammatory factors (IL-6, IL-β, COX-2, and L-gals-3) that have been implicated in promoting microglial cell activation and neuronal apoptosis involving phagocytosis pathways (Yanuck, 2019).

To begin to gain insights into the factors and pathways through which PKall and neuronal cell debris trigger inflammatory cytokines in microglial cells, we first assessed the receptors involved in this process. Our findings indicated that the increased production and release of IL-6 in response to PKall involved engagement of PAR 2 and B_2_KR, while only PAR2 was involved in promoting IL-6 production in response to neuronal cell debris. This is also evidenced by the finding that both PKall and neuronal cell debris stimulated the expression of PAR2 and B_2_KR in microglial cells. It is of interest to note here that it is likely that some of the effects we detected in response to PKall on cytokine release could be attributed to activation of B_1_-receptors expressed in microglial cells (Noda et al., 2003). Thus, it is conceivable that once microglial cells are exposed to PKall, kininogen will be cleaved to release BK. The generated BK will in turn be cleaved by the peptidases that are expressed in microglial cells to generate des-Arg^9^-BK, the B_1_-receptor ligand, which will then bind to its receptors in an autocrine manner to transduce its signal. This notion will be addressed in future studies.

Given the role autophagy plays in microglial dysregulation, we utilized SAR405, which blocks the initiation of the macroautophagy, to test the possible involvement of autophagy in the regulation of inflammation observed with PKall and the neuronal cell debris. Our findings suggest the involvement of this process in modulating the inflammatory responses to PKall and neuronal cell debris. In this regard, our data showed that inhibition of autophagy potentiated the response of PKall and neuronal cell debris markedly to generate higher levels of inflammatory cytokine IL-6, thus modifying the degree of inflammation by changing the phenotype polarization of microglial cells to produce more inflammatory cytokines (Ye et al., 2018). In light of the role of ERK1/2 in microglial neuroinflammation and neurodegenerative diseases (Sun and Nan, 2017). Our findings provided a mechanistic link for the ERK 1/2 pathway as a downstream intermediate cellular signaling mediator through which PKall and neuronal cell debris trigger the production of IL-6 in microglial cells. Taken together, these findings suggest that PKall and neuronal cell debris utilize a potential common pathway to modulate microglial cell inflammatory response that involves PAR 2 activation, autophagy and the MAPK pathway.

The findings of the current study provide novel mechanistic insights into the role of PKall in modulating microglial cell activation and dysregulation and offer new understandings of the functional scenarios through which PKall could orchestrate the generation and transmission of inflammatory mediators that promote the development of neurodegenerative diseases.

## Data Availability

The original contributions presented in the study are included in the article/Supplementary Material, further inquiries can be directed to the corresponding authors.
