# Supplementary material for: Modulation of Neuro-Inflammatory Signals in Microglia by Plasma Prekallikrein and Neuronal Cell Debris
Source: Front Pharmacol. 2021 Nov 15;12:743059. doi: 10.3389/fphar.2021.743059 (PMC8636058; doi:10.3389/fphar.2021.743059)
Supplement: Supplementary file 1 [file DataSheet2.docx]

**Supplemental Figure 1:** Coronin and IBA-1 detection in microglial cells by western blots. N9 microglial cells grown in 2 % FBS were stimulated with LPS (100ng/ml), BK (0.1 µM), PKall (2.5 ng/ml) and NCD (16.5 µg protein/ml) for 24 hours. 30 µg protein of total cell lysates were subjected to SDS-PAGE (12%), under reducing conditions and the resolved proteins were transferred to nitrocellulose membranes and immunoblotted with primary anti-coronin and anti-IBA-1 polycolonal antibodies (1/1000 dilution, Encor Biotechnologies, catalogue # AB-2722474) overnight at 4°C followed by incubating the membranes in anti-rabbit secondary antibody. The protein bands were detected using an ECL Detect Kit and visualized using Chemidoc MP imaging system (Bio-Rad, Hercules, CA). Results show that coronin and IBA-1protein expression can be detected in microglial cells in response to LPS, BK, PKall and NCD.

**Supplemental Figure 2:** Effect of SAR405 on LC3 expression profile. Bone marrow-derived macrophages (A) and Hela cells (B) were treated for 15 min with SAR405 (1 or 5 µM) in Earl’s balanced salt solution to favor autophagy prior to the incubation without (basal) or with 50 µM chloroquine (CQ) for 4 hours. 10 µg of total cell lysates were used for western blot analysis of LC3 using LC3 selective antibody (Bio-Techne NB100-2220) and b-actin. Results show that the lipidation of LC3-I in LC3II observed in macrophages was reversed upon treatment with SAR405. Similarly, in Hela cells, the lipidation of LC3-I in LC3-II, which was increased after CQ treatment compared to basal untreated cells, was significantly reversed by 1 and 5 µM SAR405. These results support the inhibition of autophagy by SAR405.


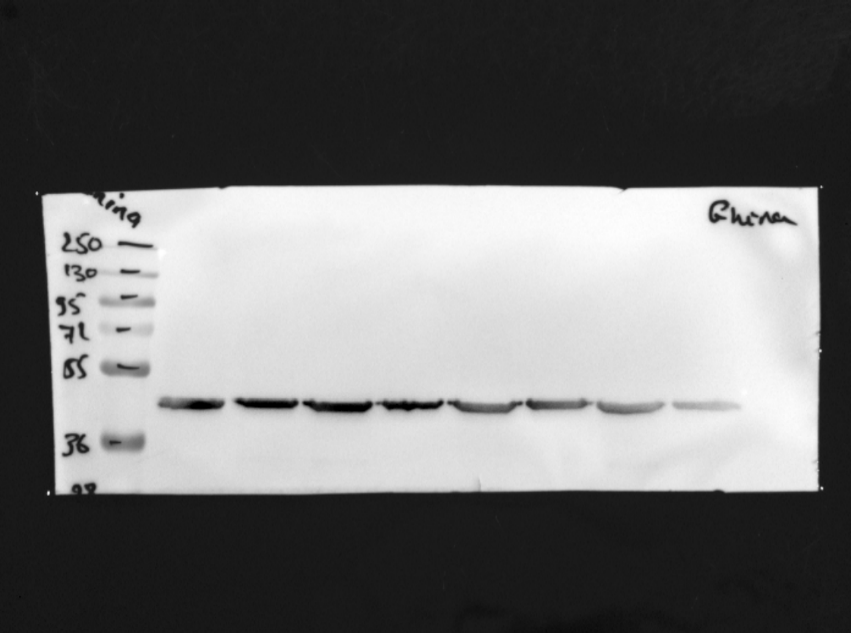

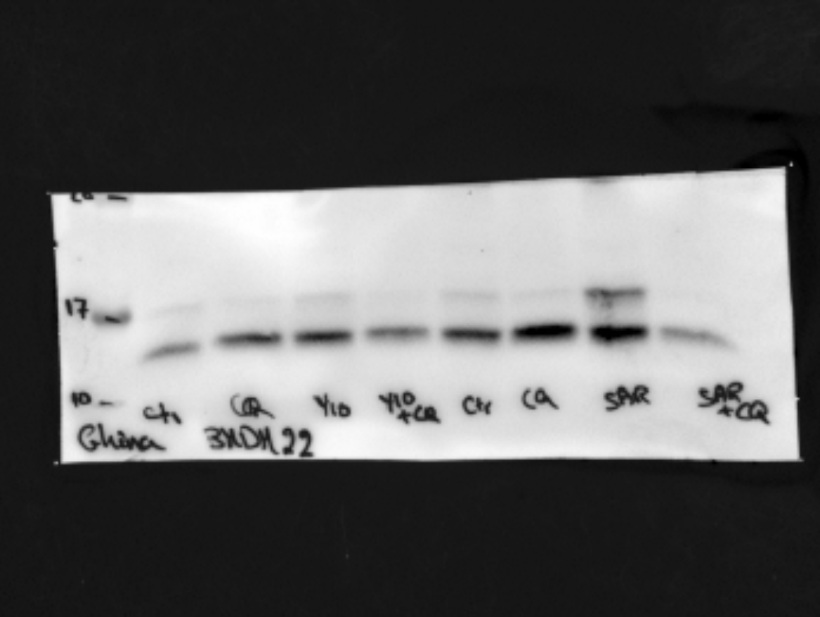


*kDa*

17

LC3-I

LC3-II

β-actin

Basal

CQ

SAR405

45

A)

B)

*kDa*

17

LC3-I

LC3-II

β-actin

CQ

SAR405

(μM)

45

0

1

5

0

1

5

Basal
